# Dimensions of artificial intelligence anxiety among employees in the age of innovation: a systematic review

**DOI:** 10.3389/fpsyg.2026.1824525

**Published:** 2026-06-19

**Authors:** Sultanah Alsudays

**Affiliations:** Business Administration Department, College of Business, Imam Mohammad Ibn Saud Islamic University (IMSIU), Riyadh, Saudi Arabia

**Keywords:** artificial intelligence (AI), artificial intelligence anxiety dimensions, Scientific Procedures and Rationales for Systematic Literature Reviews (SPAR-4-SLR), systematic literature review (SLR), Theory–Context–Characteristics–Methodology (TCCM)

## Abstract

**Introduction:**

Artificial intelligence (AI) anxiety has emerged as a significant phenomenon accompanying the digital transformation and increasing adoption of AI in workplace settings. This study aims to identify and synthesize the different dimensions of AI anxiety discussed in prior research.

**Methods:**

This systematic literature review combines the Scientific Procedures and Rationales for Systematic Literature Reviews (SPAR-4-SLR) guidelines with the Theory–Context–Characteristics–Methodology (TCCM) analytical framework. The review addresses the 3W1H research questions (What, Where, When, and How) related to AI anxiety dimensions and provides a comprehensive analysis of the theories, contexts, characteristics, and methodologies used in this research domain.

**Results:**

The findings reveal that Conservation of Resources (COR) theory and Social Cognitive Theory (SCT) are the most frequently applied theoretical perspectives. Research on AI anxiety dimensions has been conducted predominantly in China and Türkiye, particularly within the healthcare sector. General AI anxiety is the most extensively examined dimension, with numerous antecedents, mediators, moderators, and outcomes identified. In contrast, dimensions such as job replacement anxiety, AI ethics anxiety, AI learning anxiety, collective anxiety, and configuration anxiety remain relatively underexplored. Furthermore, regression analysis is the most commonly employed statistical technique in the reviewed studies.

**Discussion:**

The findings indicate a strong concentration on general AI anxiety and a limited focus on more specific dimensions across different levels of analysis. This review contributes to a comprehensive understanding of AI anxiety and its dimensions while identifying important research gaps. Practical implications for practitioners and researchers, along with study limitations and directions for future research, are also discussed.

## 1 Introduction

Dealing with artificial intelligence (AI) has become a necessity for every workplace. The world is experiencing an accelerated era of digital transformation, which affects human life and work styles (Kim et al., 2025). AI provides several advantages for business society, for instance, promoting sustainable development (Wang et al., 2025), reducing operating costs (Brynjolfsson et al., 2019; Loske and Klumpp, 2021), and enhancing service delivery (Chen et al., 2022; Guo et al., 2024). However, adopting AI requires serious attention regarding a negative phenomenon called AI anxiety. Researchers have provided several definitions of AI anxiety. For instance, Uygungil-Erdogan et al. (2025) define AI anxiety as a combination of a negative feeling and fear toward the future effects of AI. Other researchers (Kim et al., 2025) define AI anxiety as people’s distressing feelings about the rapid progress of AI technology across different life domains. Based on these definitions, it has been found that AI anxiety is a dual-component construct involving both cognitive appraisal (individuals’ evaluation of AI’s potential impacts) and affective responses (feelings of fear, worry, or distress toward AI). In addition, AI anxiety is considered an umbrella concept that includes different dimensions of anxiety, including learning, job replacement, sociotechnical blindness, AI configuration, privacy violation, bias behavior anxiety, existential risk, artificial consciousness, lack of transparency, and against ethics anxiety (Li and Huang, 2020; Wang and Wang, 2022). The dimensions of AI anxiety were discussed in previous literature by relying on several theories as a foundation for these studies. Therefore, this systematic review deeply highlights the current theories that conceptualize AI anxiety dimensions.

AI adaptation in the work context might lead to several changes. For instance, McKinsey and Company (2023) anticipates that global productivity will be affected positively by using automation and AI in the workplace. The amount of the increase in the global productivity rate will shift from 0.5% in 2023 to 3.4% in 2040. The AI contribution in this growth is expected to range from 0.1 to 0.6%. However, this vast increase is conditional upon the employee’s ability to have alternative work roles that lead to productivity levels comparable to their previous activities. In addition, according to the World Economic Forum (2025), 22% of the jobs will be affected in 2030 because of various factors, such as technological development. In all, 78 million new jobs will be created, and, simultaneously, 92 million jobs will be shrunk. Regardless of the expected substantial expansion in new employment opportunities, this transformation may increase AI anxiety among employees. Individuals’ concerns regarding their job roles during AI transformation, such as having a secure job and technical experience, are the key factors of AI anxiety (Johnson and Verdicchio, 2017). Therefore, managers should be aware of the negative impact of several dimensions of AI anxiety and implement strategies to mitigate their impact among employees by enhancing clear communication, psychological safety, and encouraging employees to develop their skills. To increase this awareness, this systematic review covers different kinds of variables, including antecedents, mediators, and moderators, and outcomes of AI anxiety dimensions. Reviewing such empirical studies not only assists employees in adapting themselves to the changes but also aids organizations in being successful for a long period.

Although there are several advantages of implementing AI in the work context, the AI anxiety dimensions remain an unexplored topic. Thus, this study aims to advance the current global literature by conducting a systematic review regarding AI anxiety dimensions in various contexts (countries and industries) to provide a comprehensive understanding of this phenomenon from diverse global perspectives with an integrated framework for future research regarding these dimensions. In addition, as this study concentrates on synthesizing empirically examined relationships, including mediating and moderating mechanisms, quantitative studies are appropriate in this situation. Identifying the statistical techniques that measure these relationships should be explored.

Based on the study objective, the following questions will be highlighted:

- RQ1: How do current theories conceptualize AI anxiety dimensions?
- RQ2: What are the characteristics of the key variables associated with different dimensions of AI anxiety, including antecedents, mediating, moderating variables, and outcomes?
- RQ3: What are the contexts (countries and industries) in which empirical studies on AI anxiety dimensions have been conducted so far?
- RQ4: What are the main quantitative statistical techniques that have been applied in the empirical studies of AI anxiety dimensions

To provide a comprehensive answer to the research questions, a systematic literature review (SLR) is employed (Snyder, 2019), which examines AI anxiety dimensions across multiple sectors, including theories, characteristics of the key variables associated with different dimensions of AI anxiety, research context, and method in depth. Few emerging studies (e.g., Rizkina et al. 2025) have attempted to apply a systematic review for AI anxiety; thus, to date, this systematic review is among the first that applies a new rigorous protocol called the Scientific Procedures and Rationales for Systematic Literature Reviews (SPAR-4-SLR) protocol (Paul et al., 2021). In addition, this review uses the Theory–Context–Characteristics–Methodology (TCCM) analytical framework (Paul and Rosado-Serrano, 2019) to analyze the literature outcomes. Furthermore, this study provides a comprehensive review regarding the antecedents and consequences by relying on several published articles in Web of Science (WOS) and Scopus indexed in SSCI: Social Sciences Citation Index (SSCI) and Science Citation Index Expanded (SCIE). In this study, 31 articles were selected from the WOS and Scopus databases.

In addition to investigating a critical gap in the literature regarding the higher-order concept of AI anxiety and its dimensions, this study contributes to the current literature by providing an integrated analysis of AI anxiety dimensions in several work contexts. This will provide a clear picture of what the employees encounter with the digital transformation to AI. Understanding these concerns will lead to generating valuable implications not only for researchers but also for practitioners who deal with AI in the work environment to mitigate its negative effects.

Furthermore, this study makes several valuable contributions to the research society. First, it proposes an extensive framework regarding AI anxiety dimensions. Second, it highlights, in-depth, AI anxiety’s antecedents and consequences, which guide future research and practitioners in this critical phenomenon. Finally, to enhance our understanding of the several dimensions of AI anxiety, valuable recommendations for future research were suggested, including conducting empirical studies to investigate the antecedents and consequences of several AI anxiety dimensions in new contexts and applying comparative studies between different cultural contexts.

## 2 Systematic review methodology

To integrate nascent research fields, SLR is a useful tool (Paul et al., 2021; Paul and Criado, 2020). A domain-based review approach will be used as well to concentrate on highlighting a specific research topic through the TCCM analytical framework (Paul and Rosado-Serrano, 2019), which classifies the outcomes of the literature review and guides future research. There are four key factors in the TCCM analytical framework. The first factor is Theory (T), which includes a selection of concepts that clarify the constructs’ relationship for a specific phenomenon. The second factor is Context (C), which reflects the research setting and context, such as the industry type. The third factor is Characteristics, which refers to the construct types used in each study (e.g., antecedents, consequences, mediators, and moderators). The last factor is Methods (M), which discusses the methodological part of each study (e.g., the sample type, the study’s measure, and its analysis) (Paul et al., 2024). In addition, to foster methodological rigor, clarity, and theoretical richness in this SLR, the SPAR-4-SLR technique was employed. This strong combination of the TCCM analytical framework and SPAR-4-SLR to discuss a new phenomenon is considered a solid foundation for conducting SLR.

### 2.1 SPAR-4-SLR protocol

**Figure 1:**
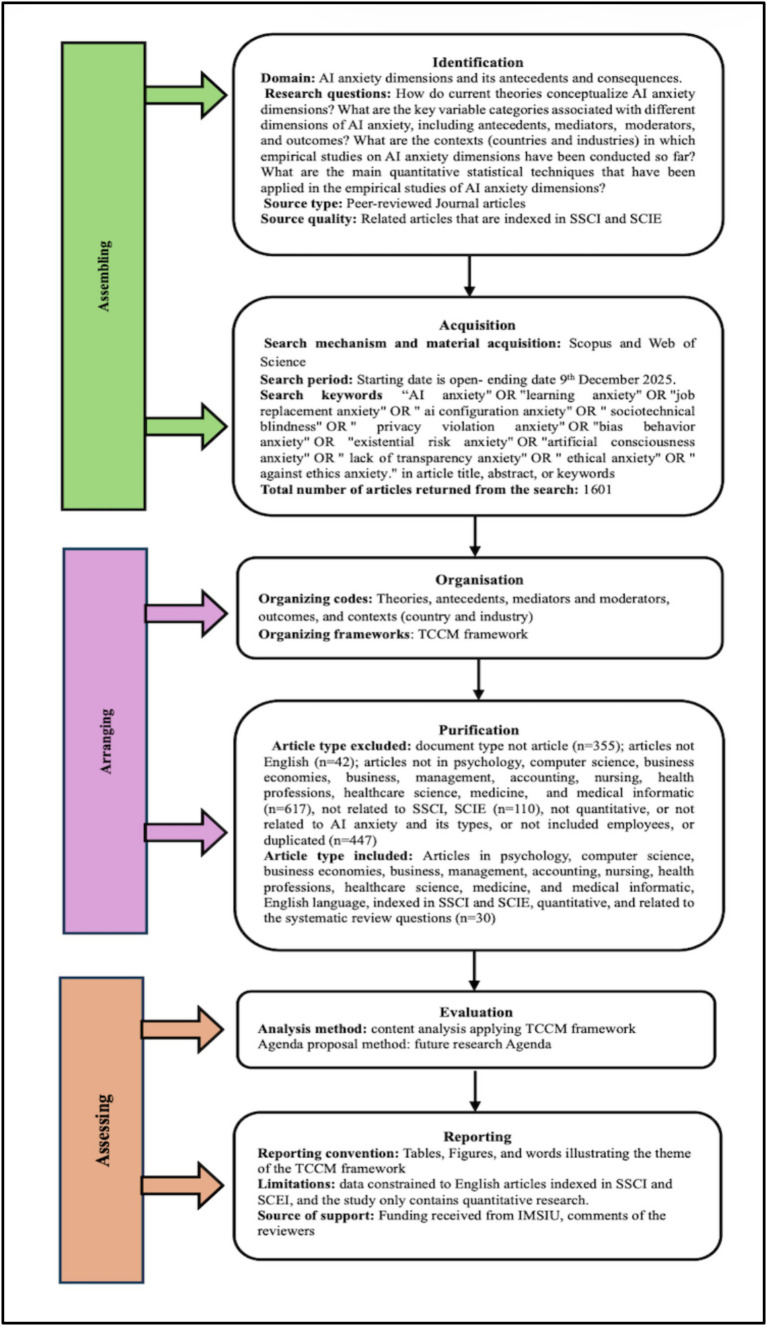
SPAR-4-SLR protocol.

Three main stages should be followed to apply the SPAR-4-SLR technique (Paul et al., 2021), and each stage includes two substages. Stage one is called assembling and includes identifying and acquiring literature. Stage two is called arranging and includes organizing and purifying literature. The last stage is called assessing, which includes evaluating and reporting literature (Sharma et al., 2023) (see Figure 1).

#### 2.1.1 Assembling

##### 2.1.1.1 Identification

Peer-reviewed academic journal articles have high quality compared to other types of literature sources (Paul et al., 2021). Thus, this study includes only this kind of literature. In addition, two high-prestige and reliable databases, namely, WOS and Scopus, were selected to collect the required published articles that belong to two main indices: SSCI and SCIE.

##### 2.1.1.2 Acquisition

To enhance the value of this SLR, the peer-reviewed academic articles that were collected were limited only to the WOS and Scopus databases. The beginning search time period was opened because of the novelty of the topic, AI anxiety, and the end search period was 9 December 2025. To increase the accuracy of the related articles, the technique adopted to select the search terms is based on SPAR-4-SLR, and these keywords should reflect the systematic review research questions and were selected through a brainstorming session with some academic experts. The search includes specific keywords that concentrates on AI anxiety and its dimensions: “AI anxiety” OR “learning anxiety” OR “job replacement anxiety” OR “AI configuration anxiety” OR “sociotechnical blindness” OR “privacy violation anxiety” OR “bias behavior anxiety” OR “existential risk anxiety” OR “artificial consciousness anxiety” OR “lack of transparency anxiety” OR “ethical anxiety” OR “against ethics anxiety” in article title, abstract, or keywords. By focusing on these keywords, the research questions can be answered because these terms are mostly measured by applying a quantitative approach and investigating them will provide answers for the research questions. The search resulted in 1,601 articles.

#### 2.1.2 Arranging

##### 2.1.2.1 Organization

Figure 1 shows the key steps for organizing this search process in this SLR. Furthermore, the analytical framework was applied to get several related articles.

##### 2.1.2.2 Purification

The literature search was conducted over multiple sessions across the selected databases (WOS and Scopus) to ensure comprehensiveness and accuracy. Each database was searched systematically, and the results were recorded and managed consistently throughout the process. The final search was completed on 9 December 2025, and all retrieved records were consolidated for screening. This approach enhances the transparency and reproducibility of the review process.

The total number of preliminary research articles was 1,601. Several filtration stages were used. First, the number of excluded articles that were not classified as research articles was 355. Second, the number of excluded articles that were not in English was 42. Third, the number of excluded articles that were not in the following domains—“psychology, computer science, business economics, business, management, accounting, nursing, health professions, healthcare science, medicine, and medical informatics”—was 617. Fourth, the number of excluded articles that were not related to SSCI and SCIE was 110. Finally, the number of excluded articles that were not quantitative, or not related to AI anxiety and its dimensions, or did not include employees, or were duplicated was 447. Consequently, 30 articles that met the required criteria were included. The total sample size across all included studies is 11,638, with individual study samples ranging from 150 to 1,606 participants.

#### 2.1.3 Assessing

##### 2.1.3.1 Evaluation

The analysis tool adapted in this SLR is the TCCM analytical framework to assess the studies’ results.

##### 2.1.3.2 Reporting

Based on the evaluation process, several tools were used, such as tables and figures, to summarize the main findings.

### 2.2 Review findings based on TCCM analytical framework

**Figure 2:**
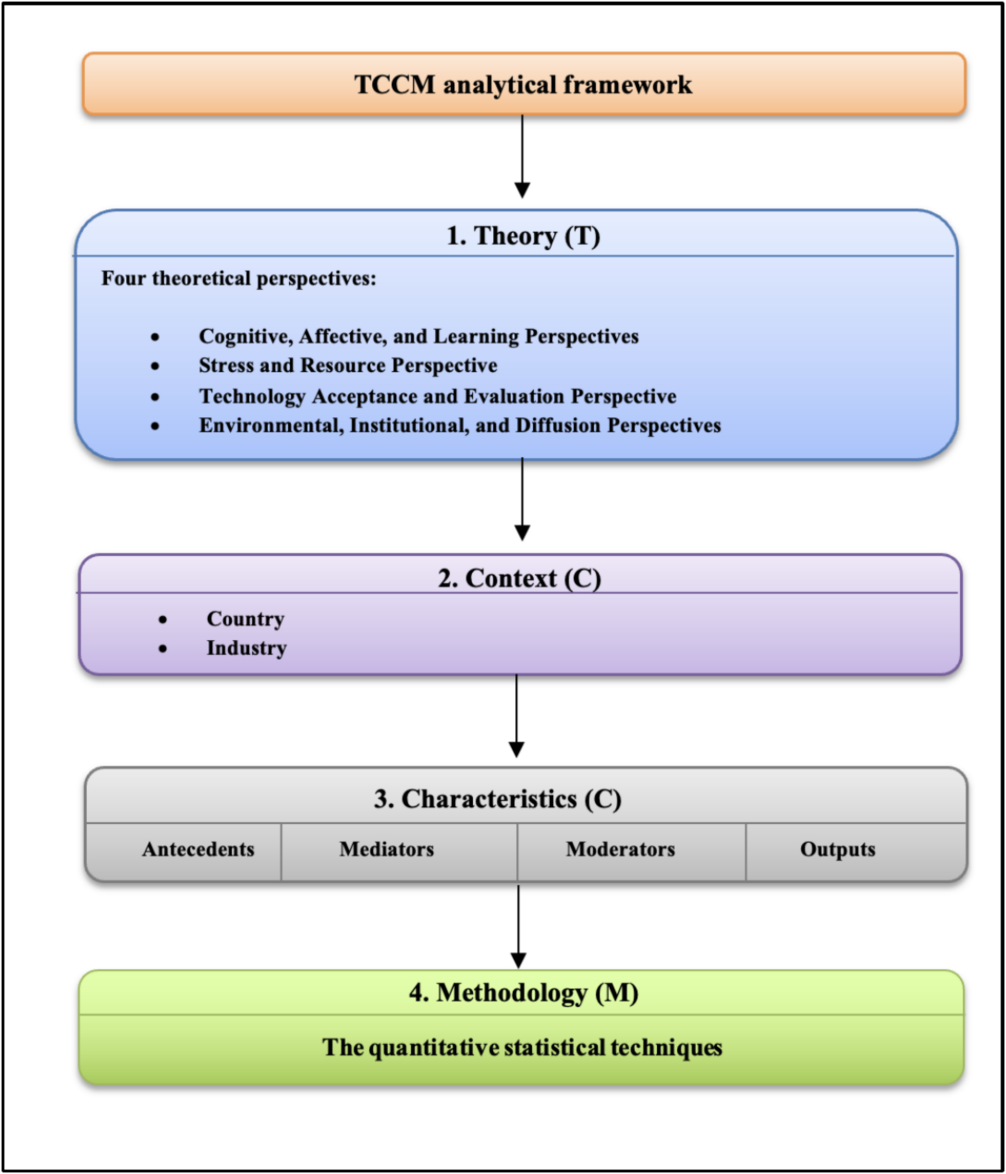
Hierarchical organization of the TCCM analytical framework.

The findings of the collected articles in this SLR are analyzed using the TCCM analytical framework. This section will provide a deep discussion in terms of the theories, context, characteristics, and methods used in the collected articles. Figure 2 shows the hierarchical organization of the TCCM Analytical Framework in the present study.

Before proceeding to discuss each component of the TCCM analytical framework, Table 1 summarizes all the articles according to the analytical framework. The gray shaded cells under characteristics (C) represent the role of the focal variable (the dimensions of AI anxiety) in each study, while the remaining cells under characteristics (C) represent the variables associated with each focal construct, as reported in the original studies.

**Table 1:** TCCM analytical framework.

Author/s	TCCM analytical framework							
	Theory (T)	Context (C)		Characteristics (C)				Methods (M)
		Country	Industry	Antecedents	Mediators	Moderators	Outcomes	
Chen and Zhao (2025)	Integrated fear acquisition theory (IFAT)	China	Several sectors	Job replacement anxiety	-	-	Job optimism	Regression technique
Zhou et al. (2025)	Social cognitive theory (CST)	China	Healthcare sector	Learning anxiety	-	-	Continuous Adoption of AI technology	Structural equation modeling technique (SEM)
					-	-	AI trust	
					-	-	Organizational trust	
					AI trust	-	Continuous adoption of AI technology	
					Organizational trust			
				Job substitution anxiety	-	-	AI trust	
					-	-	Organizational trust	
					AI trust	-	Continuous adoption of AI technology	
					Organizational trust			
Qian et al. (2025)	Conservation of resources theory (COR)	China	Technological sectors	AI usage	-	-	Job replacement anxiety	Path and bootstrap resampling technique
				Job replacement anxiety	-	-	Proactive skill development	
				AI usage	Job replacement anxiety	Learning goal orientation	Proactive skill development	
Tarsuslu et al. (2025)	-	Türkiye	Healthcare sector	Digital leadership	-	-	General AI anxiety	Regression technique
				AI attitude	-	-		
				Digital leadership	AI attitude	-		
Sha et al. (2025)	Self-control theory	China	E-commerce sector	General AI anxiety	-	-	Deviant behavior	Process model technique
					-	-	Emotional exhaustion	
					Emotional exhaustion	-	Deviant behavior	
					Emotional exhaustion	Leader support	Deviant behavior	
Fan et al. (2025)	Job Demand–Control model (JDC) model and emotional cognitive evaluation theory	Several countries	Healthcare sector	Perceived substitution of AI	-	-	General AI anxiety	SEM
				General AI anxiety	-	-	Innovate work behavior	
					-	-	Human-AI cooperation intention	
				Perceived substitution of AI	General AI anxiety	-	Innovate work behavior	
				Perceived substitution of AI	General AI anxiety	-	Innovate work behavior	
					Human-AI cooperation intention			
Chen et al. (2025)	COR	China	Manufacturing sector	Job replacement anxiety	-	-	Work passion	Partial least squares structural equation modeling (PLS-SEM) technique
					-	-	Emotional exhaustion	
					Emotional exhaustion	-	Work passion	
					-	Service-oriented leadership	Emotional exhaustion	
				Learning anxiety	-	-	Work passion	
					-	-	Emotional exhaustion	
					Emotional exhaustion	-	Work passion	
					-	Learning goal orientation	Emotional exhaustion	
Özçevik Subaşi et al. (2025)	-	Türkiye	Healthcare sector	General attitudes toward AI scale	-	-	General AI anxiety	Regression and Pearson’s correlation technique
Uygungil-Erdogan et al. (2025)	Uncanny valley theory	Türkiye	SMEs sector	General AI anxiety	-	-	Quiet quitting	Bootstrapping calculation technique
					Quiet quitting	-	Turnover intention	
Li et al. (2024)	JDC model and SCT	China	Healthcare sector	Medical explainable AI	-	-	General AI anxiety	SEM
				General AI anxiety	-	-	Innovation behavior	
				Medical explainable AI	General AI anxiety	-	Innovation behavior	
Feng et al. (2025)	COR and unified theory of acceptance and use of technology (UTAUT)	China	Service sector	General AI anxiety	-	-	Life satisfaction	Regression technique
					Negative emotions	-	Life satisfaction	
					-	Social support	Negative emotions	
Alqaifi and Tengilimoglu (2025)	UTAUT	Türkiye	Healthcare sector	General AI anxiety	-	-	Employee intention to adopt AI	PLS-SEM
Xu et al. (2025)	COR	China	Several sectors	Perceived Risk of Unemployment	-	-	General AI anxiety	Regression technique
				General AI anxiety	-	-	Depression	
				Perceived risk of unemployment	General AI anxiety	Mindfulness	Depression	
				General AI anxiety	-	Mindfulness	Depression	
Suseno et al. (2023)	Tripartite model of attitude (TMA) and SCT	China	Human resource sector	General AI anxiety	-	-	Change readiness for AI adoption	Regression technique
					-	High-performance work systems	Change readiness for AI adoption	
Chang et al. (2024)	Affective events theory (AET) and challenge–hindrance stressor framework (CHSF)	China	-	AI-driven hindrance technology stressors	-	-	General AI anxiety	Regression and Process macro technique
				General AI anxiety	-	-	AI adoption intention	
				AI-driven hindrance technology stressors	General AI anxiety	-	AI adoption intention	
				AI-driven hindrance technology stressors	-	Technical self-efficacy	General AI anxiety	
				AI-driven hindrance technology stressors	General AI anxiety	Technical self-efficacy	AI adoption intention	
Huo et al. (2023)	Stimulus-organism-response framework (SOR)	China	Healthcare sector	Medical staff participation	-	-	General AI anxiety	SEM
				General AI anxiety	-	-	Acceptance of medical AI-IDT	
				Medical staff participation	General AI anxiety	-	Acceptance of medical AI-ADT	
				Medical staff participation	-	Speciesism	General AI anxiety	
Wu et al. (2025)	UTAUT	China	Construction sector	General AI anxiety	-	-	Users’ usage behavior of AI	SEM
					-	-	Users’ effort expectancy of AI	
					-	-	Social influence in AI adoption	
Nasaj et al. (2026)	SCT	Europe	Construction sector	Collective AI anxiety	-	-	Team innovative behaviors	SEM
					Problem-focused coping	-	Team innovative behaviors	
					Emotion-focused coping	-	Team innovative behaviors	
					Relation-focused coping	-	Team innovative behaviors	
Tao et al. (2025)	SCT	China	Marketing sector	AI privacy anxiety	-	-	AI trust	Regression technique
					-	-	Intention to use AI-generated content	
				AI bias anxiety	-	-	AI trust	
					-	-	Intention to use AI-generated content	
				AI opacity anxiety	-	-	AI trust	
					-	-	Intention to use AI generated content	
				General AI anxiety	AI trust	-	Intention to use AI-generated content	
Nguyen et al. (2025)	Diffusion of innovations theory (DOI)	Vietnam	Marketing sector	General AI anxiety	-	-	AI Adoption intention	SEM
Weber et al. (2024)	-	-	Healthcare sector	AI knowledge	-	Job replacement anxiety	Intention to use AI	Regression technique
Maldonado-Canca et al. (2024)	UTAUT	Spain	Several sectors	General AI anxiety	-	-	Intention to adopt AI tools	PLS-SEM
Modliński et al. (2024)	Technology acceptance model (TAM)	Poland	Cultural sector	Job replacement anxiety	-	-	Reactions to human-machine trans roles conflict	Regression technique
Jo and Park (2025)	-	Korea	Managerial sector	Anthropomorphism of generative AI	-	-	Job replacement anxiety	PLS-SEM
				Personalization of generative AI	-	-		
				Skepticism toward generative AI	-	-		
				The self-learning capability of generative AI	-	Skepticism toward GAI		
				Anthropomorphism of generative AI	-	Skepticism toward GAI		
Wang and Zhang (2025)	DOI	China	Retailing sector	Digital employee adoption	-	Ethical anxiety	Exploitative green innovation	Bootstrapping technique
				Digital employee adoption	-		Exploratory green innovation	
Konuk et al. (2023)	COR	Türkiye	Call center industry	Learning anxiety	-	-	Psychological wellbeing	Regression technique
Soomro et al. (2024)	AET	Pakistan	Manufacturing sector	-	-	-	Employees’ resistance to adopting AI technology	PLS-SEM
				General AI anxiety	-	-	Unfeeling of employee wellbeing	
					AI adoption	-	Unfeeling of employee wellbeing	
					-	Organizational support	Unfeeling of employee wellbeing	
Dhilipan et al. (2025)	The stress and coping theory	India	Several sectors	General AI anxiety	-	-	Wellbeing	Regression technique
					-	-	Performance	
					-	-	Career development	
					-	AI anxiety coping strategies	AI anxiety outcomes (Wellbeing, performance, career development)	
Seven et al. (2025)	-	Türkiye	Healthcare sector	Learning anxiety	-	-	Positive attitudes to AI	SEM
					-	-	Negative attitudes to AI	
				AI configuration anxiety	-	-	Positive attitudes to AI	
Chou et al. (2025)	SCT	Taiwan	Healthcare sector	AI self-efficacy	-	-	General AI anxiety	PLS-SEM and artificial neural network (ANN) technique
				General AI anxiety	-	-	Intention to adopt the AI system	

The gray shaded cells under characteristics (C) represent the role of the focal variable (the dimensions of AI anxiety) in each study.

#### 2.2.1 Theory (T)

**Figure 3:**
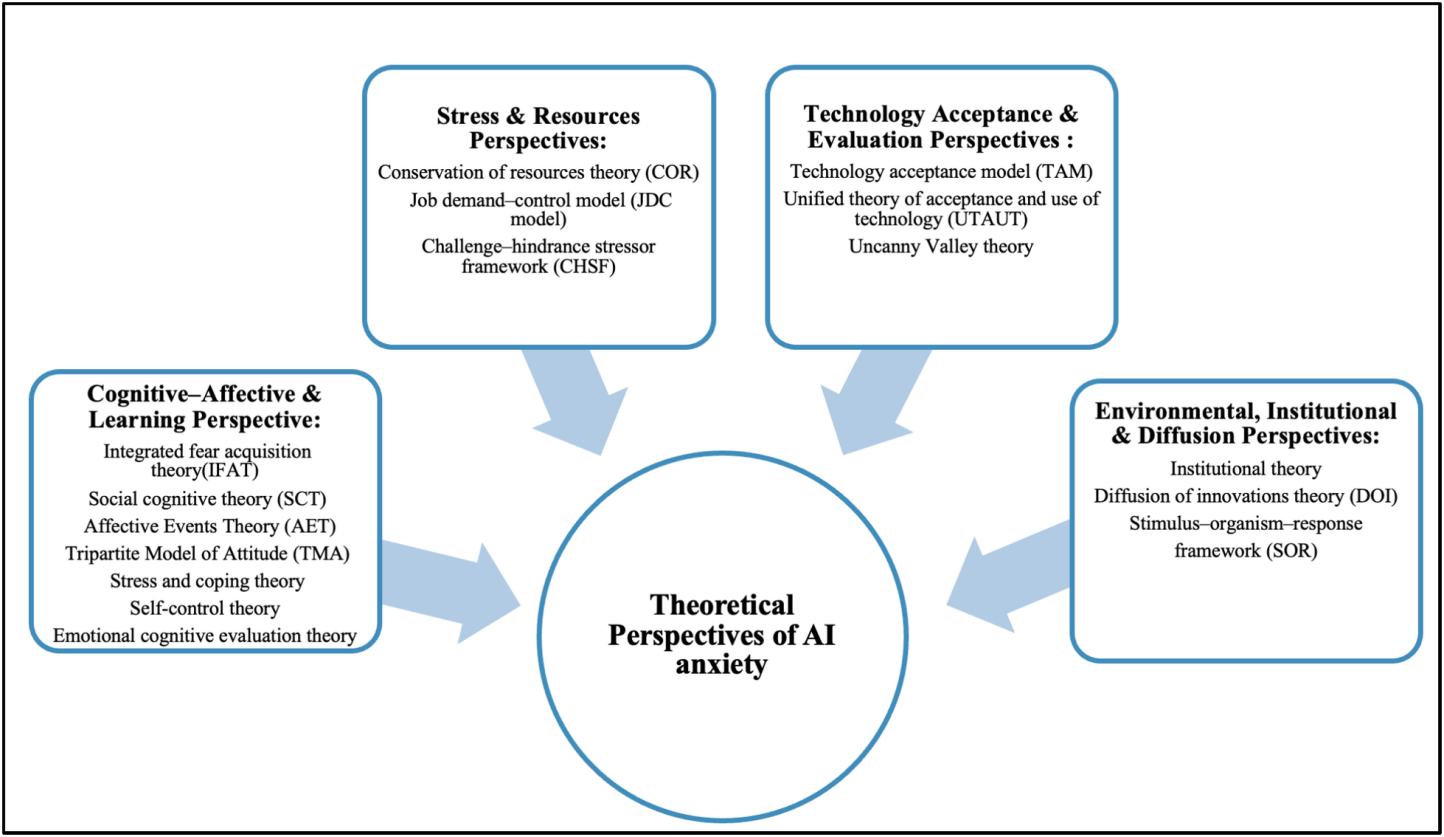
Theoretical perspectives on AI anxiety.

This section complements the TCCM synthesis by organizing the individuals’ negative feelings and fear toward AI development, which were discussed in previous research from several broader theoretical perspectives. Figure 3 shows these perspectives: First, cognitive, affective, and learning perspectives. Second, a stress and resource perspective. Third, a technology acceptance and evaluation perspective. Finally, environmental, institutional, and diffusion perspectives. These perspectives illustrate how theories are applied across different contexts and linked to key constructs examined in the studies.

##### 2.2.1.1 Cognitive, affective, and learning perspectives

In light of cognitive, affective, and learning perspectives, the integrated fear acquisition theory (IFAT) (Li and Huang, 2020), social cognitive theory (SCT) (Bandura, 1977, 1986), affective event theory (AET) (Weiss and Cropanzano, 1996), tripartite model of attitude (TMA) (Ajzen, 2001, 2005), stress and coping theory (Lazarus and Folkman, 1984), stress and coping theory (Lazarus and Folkman, 1984), self-control theory (Gottfredson and Hirschi 1990), and emotional cognitive evaluation theory (Lazarus, 1991) clarify the emotional and mental responses of individuals toward AI anxiety. Li and Huang (2020) suggest that the causes of AI anxiety can be identified by using the IFAT as fear is considered a cognitive source. In addition, the SCT highlights that an individual’s behavior is derived from the interactional relationship among the environment, person, and behavior itself. In the AI anxiety context, the SCT is also used to reflect the relationships between the environmental factor (e.g., support from others), self-derived factor (e.g., feeling of AI anxiety), and behavioral factors (e.g., intention to use AI) (Chou et al., 2025), while the AET reflects the significance of events within the work context in workforce behaviors and emotions. Thus, employing AI within the work context is a significant event that can lead to feelings of AI anxiety among employees (Soomro et al., 2024). Prior research also applied the TMA, which suggests that attitude comprises three factors: cognitive, affective, and behavioral elements (Ajzen, 2001, 2005; Eagly, 1993; Piderit, 2000; Rosenberg, 1960), although the alignment between the three factors is not a must. For instance, individuals might have positive cognitive and affective attitudes regarding AI. However, when they encounter a situation that requires them to learn new knowledge about AI, they might have a negative behavioral attitude toward learning new knowledge (Suseno et al., 2023). In addition, stress and coping theory suggests that stress is derived from individuals’ cognitive evaluation of the situations and how they can deal with them, rather than from the situation per se. Thus, this theory was used to explain the effect of AI anxiety levels on coping methods. If people feel high AI anxiety, they may engage in problem-focused coping, which will positively affect the adoption process with the situation they face. However, if the level of AI anxiety is extremely high, a negative response will occur (Dhilipan et al., 2025). Prior research also uses the self-control theory to explain AI anxiety. According to the self-control theory, when people feel low self-control, they may adopt unfavorable behaviors (Gottfredson and Hirschi, 1990). For instance, AI anxiety negatively affects the individual’s ability to have self-control, which leads to unfavorable behaviors (Sha et al., 2025). The final theory in this perspective is emotional cognitive evaluation (Lazarus, 1991). This theory argues that individuals’ feelings affect the behaviors that they will adopt (Lazarus, 1991). For instance, when people feel AI anxiety, they will adopt certain behaviors that help them to avoid any risks (Fan et al., 2025). Table 2 lists the theories applied within the cognitive, affective, and learning perspectives, along with the corresponding articles in which each theory was used.

**Table 2:** Prominent countries.

Country/Region			Number of studies	Example
Single-country study	Multi-country studies	Region		
China	-	-	14	Chen and Zhao (2025); Zhou et al. (2025); Qian et al. (2025); Sha et al. (2025); Chen et al. (2025); Feng et al. (2025); Li et al. (2024); Xu et al. (2025); Suseno et al. (2023); Chang et al. (2024); Wu et al. (2025); Tao et al., (2025); Huo et al. (2023); Wang and Zhang (2025)
Türkiye	-	-	6	Tarsuslu et al. (2025); Özçevik Subaşi et al. (2025); Uygungil-Erdogan et al. (2025); Alqaifi and Tengilimoglu (2025); Konuk et al. (2023); Seven et al. (2025)
Vietnam	-	-	1	Nguyen et al. (2025)
Spain	-	-	1	Maldonado-Canca et al. (2024)
Poland	-	-	1	Modliński et al. (2024)
Korea	-	-	1	Jo and Park (2025)
Pakistan	-	-	1	Soomro et al. (2024)
India	-	-	1	Dhilipan et al. (2025)
Taiwan	-	-	1	Chou et al. (2025)
-	-	Europe	1	Nasaj et al. (2026)
-	China, Kazakhstan, Malaysia, UK, USA, and New Zealand	-	1	Fan et al. (2025)
-			1	Weber et al. (2024)

##### 2.2.1.2 Stress and resource perspective

From a stress and resource perspective, the conservation of resources theory (COR) (Hobfoll, 1989), the job demand–control model (JDC) model (Karasek, 1979), and the challenge–hindrance stressor framework (CHSF) (Cavanaugh et al., 2000) clarify the threatened feeling of individuals from AI. For instance, the CORT indicates that when people expect to lose their resources, they begin to feel stress, and this feeling negatively affects their proactive behavior and protects the resources they have (Hobfoll, 1989). In terms of AI anxiety, people feel they might lose their resources as a result of adopting AI, which negatively affects their desire to stay within the organization (Koo et al., 2021). Furthermore, the JDC model explains how individuals’ wellbeing is affected negatively by the stress that they encounter based on their job demand and job control (Karasek, 1979). According to this model, AI anxiety feelings may increase when an imbalance exists between job demand and job control because of AI adoption. Regarding CHSF, it argues that job stress might affect employees negatively or positively, depending on the kind of stress. Challenge stressors affect employees positively, while hindrance stressors affect employees negatively (Cavanaugh et al., 2000). Thus, if employees deal with AI as a challenge stressor, this will reduce AI anxiety (Li et al., 2020). However, if they deal with AI as hindrance stressors, this will increase AI anxiety (Maier et al., 2021).

##### 2.2.1.3 Technology acceptance and evaluation perspective

In terms of the technology acceptance and evaluation perspective, the technology acceptance model (TAM) (Davis, 1989), the unified theory of acceptance and use of technology (UTAUT) (Venkatesh et al., 2003), and the uncanny valley theory (Mori, 2012) explain the reasons behind individuals’ acceptance or rejection of AI. For instance, the TAM indicates that technology adoption decisions from individual perspectives rely on the extent of the usefulness and the simplicity of the technology (Davis, 1989). Thus, AI anxiety might decrease the usefulness and the simplicity of using AI, which leads to negative reactions (Modliński et al., 2024). In addition, the UTAUT clarifies that performance expectancy factors, effort expectancy factors, social influence, and facilitating conditions are the key factors that affect an individual’s intention to use technology (Venkatesh et al., 2003). Based on the UTAUT, AI anxiety is found to reduce the feeling of AI usefulness, which negatively affects users’ performance expectations of AI technology (Wu et al., 2025). Regarding the uncanny valley theory (Mori, 2012), it reflects people’s reaction toward anthropomorphic technologies, such as robots. Thus, in light of this theory, AI anxiety might increase because of the misunderstanding of AI, which leads to negative outcomes (Uygungil-Erdogan et al., 2025).

##### 2.2.1.4 Environmental, institutional, and diffusion perspectives

Finally, broad clarifications for the social and environmental impact of AI are provided from the view of the diffusion of innovations theory (DOI) (Rogers, 2003) and the stimulus-organism-response framework (SOR) (Mehrabian and Russell, 1974). The DOI argues that specific factors, including institutional pressures, organizational culture, and personal characteristics, impact individuals’ reactions toward new technologies (Rogers, 2003). In addition, Nguyen et al. (2025) indicate that the DOI clarifies the effect of psychological barriers on applying new technology. As AI anxiety is considered a psychological barrier, it might negatively affect AI adoption (Nguyen et al., 2025). In addition, the SOR suggests that environmental elements (stimuli), such as the conditions of the work environment, impact the feelings of individuals (the organism) through including two paths, cognitive and affective, which, in turn, shape their reactions (Mehrabian and Russell, 1974). In light of the SOR, the transformation toward AI leads to a negative effect (AI anxiety), which reduces the desire of individuals to participate in positive behaviors (Fisher et al., 2013; Kwak et al., 2022; Uygungil-Erdogan et al., 2025).

Drawing on existing synthesized theoretical perspectives, AI anxiety can be investigated from several theoretical lenses. Cognitive, affective, and learning perspectives concentrate on individuals’ emotional and mental responses in terms of AI anxiety. Stress and resource perspectives reflect how individuals’ reactions may transform into feeling threatened stems from adopting AI. The technology acceptance and evaluation perspective highlights an individual’s intention to accept or reject AI adoption. Finally, the environmental, institutional, and diffusion perspective focuses on the broad social and environmental impact of AI. Overall, these perspectives provide an integrated understanding of the reviewed literature in line with the TCCM analytical framework. Figure 3 categorizes the theories based on the least applied to the most applied in the literature.

**Figure 4:**
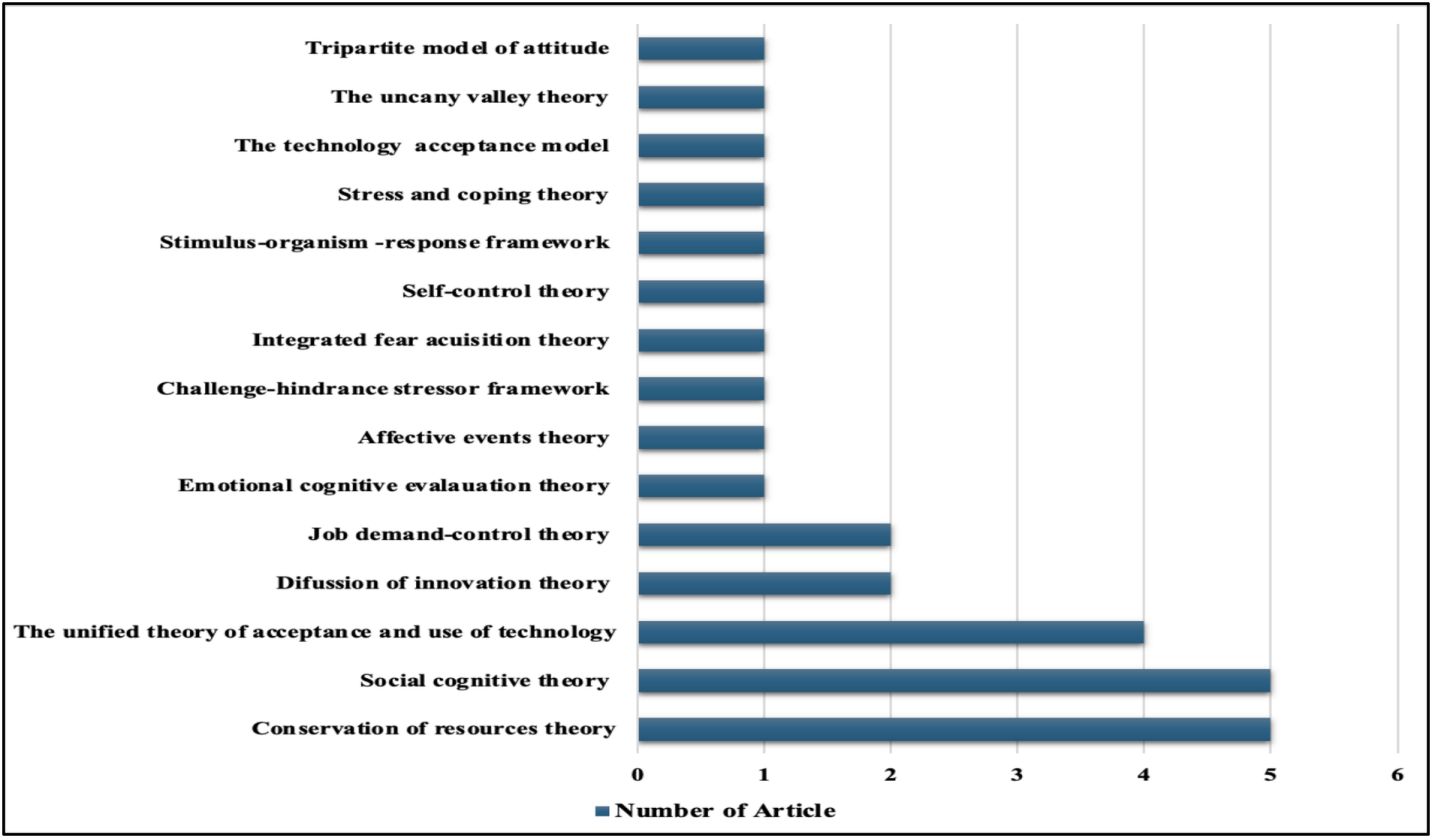
Frequency of theories applied in the reviewed articles.

Taken together, these four perspectives provide an integrated understanding of the reviewed literature in line with the TCCM analytical framework. Figure 4 categorizes the theories based on the least applied to the most applied in the literature.

#### 2.2.2 Context (C)

This section complements the TCCM synthesis by discussing the contexts (country and industry) in which AI anxiety happens, reflecting how situational and environmental factors shape theoretical perspectives, methodological choices, and key constructs.

##### 2.2.2.1 Countries

Table 2 summarizes all the included articles based on country/region. China has the highest number of publications (14) regarding AI anxiety, followed by Türkiye (6). Seven studies were distributed equally among Vietnam, Spain, Poland, Korea, Pakistan, India, and Taiwan. In addition, China, Kazakhstan, Malaysia, the UK, the USA, and New Zealand each have only one study. However, one of the included articles did not report the country context.

##### 2.2.2.2 Industry

Several industries have previously been identified by research as areas of concern regarding AI anxiety. Table 3 shows that the healthcare industry has the highest number of publications (10). Then, three industries, including manufacturing, construction, and marketing, have two publications each, while seven industries, including small and medium enterprises, service, culture, e-commerce, and high technology, have one publication each. In addition, there are four multi-industry published articles (Dhilipan et al., 2025; Xu et al., 2025; Maldonado-Canca et al., 2024; Li et al., 2024). Three articles did not report the type of industry.

**Table 3:** Prominent industries.

Industry		Number of studies	Example
Single industry study	Multi-industry studies		
Healthcare sector	-	11	Tarsuslu et al. (2025); Zhou et al. (2025); Fan et al. (2025); Özçevik Subaşi et al. (2025); Li et al. (2024); Alqaifi and Tengilimoglu (2025); Huo et al. (2023); Weber et al. (2024); Seven et al. (2025); Chou et al. (2025)
Manufacturing sector	-	2	Soomro et al. (2024); Chen et al. (2025)
Construction sector	-	2	Wu et al. (2025); Nasaj et al. (2026)
Marketing sector	-	2	Tao et al. (2025); Nguyen et al. (2025)
Small and medium enterprises sector	-	1	Uygungil-Erdogan et al. (2025)
Service sector	-	1	Feng et al. (2025)
Cultural sector	-	1	Modliński et al. (2024)
Online retailing sector	-	1	Wang and Zhang (2025)
Call center industry	-	1	Konuk et al. (2023)
E-commerce sector	-	1	Sha et al. (2025)
High-technological sector	-	1	Qian et al. (2025)
-	Information technology, Business process outsourcing, Knowledge process outsourcing, Manufacturing, Healthcare, Education, and Finance	1	Dhilipan et al. (2025)
-	Consulting, Management, Healthcare, Technology/Research and Development, Education, Marketing/Public Relations, Legal services, Architecture, and Journalism	1	Xu et al. (2025)
-	Commerce, Manufacturing Technological services, Culture, and Health	1	Maldonado-Canca et al. (2024)
-	Production, Building Communication, Technology, Logistics, and Legal.	1	Li et al. (2024)
Not reported		3	Chen and Zhao (2025); Chang et al. (2024); Suseno et al. (2023)

These contextual insights feed into the TCCM analytical framework, connecting variations in settings and populations to the key constructs, theories, and methods analyzed in the review.

#### 2.2.3 Characteristics (C)

This section complements the TCCM synthesis by discussing the characteristics of the key variables studied in relation to AI anxiety dimensions, highlighting antecedents, mediators, moderators, and outcomes.

The constructs’ characteristics examined across the reviewed studies reveal several important patterns. First, antecedents of AI anxiety dimensions are distributed across multiple levels, including organizational (e.g., leadership), team, and individual factors (e.g., self-efficacy and general attitudes toward AI). This finding reflects the multi-level and context-dependent nature of AI anxiety dimensions. Second, the literature demonstrates variation in the types of mediating mechanisms through which AI anxiety dimensions operate. Most of the mediators are cognitive and emotional in nature, including perceptions, attitudes, and emotional responses such as exhaustion. This finding indicates that AI-related issues are typically processed via internal appraisal and affective reactions before affecting outcomes. Third, moderators are less frequently examined in the reviewed literature. They are often contextual or relational, such as leadership styles and social support. This finding clarifies that the impact of AI anxiety dimensions is contingent on surrounding conditions. Fourth, the outcomes associated with AI anxiety dimensions can be broadly categorized into behavioral and strain-related outcomes. Behavioral outcomes include adoption, innovation, and cooperation, whereas strain-related outcomes include emotional exhaustion, withdrawal, and reduced work engagement. Notably, these outcomes vary across different dimensions of AI anxiety, with general AI anxiety more often linked to behavioral responses, while other AI anxiety dimensions are more strongly associated with strain outcomes. Overall, these patterns suggest that the selection and role of constructs in AI anxiety research are not arbitrary but systematically aligned with the conceptual nature of each dimension of AI anxiety.

The following subsections further elaborate on these patterns by addressing variations in the prevalence of AI anxiety dimensions and their associations with antecedents, mediators, moderators, and outcomes.

##### 2.2.3.1 Antecedents variables

The antecedents are classified by the AI anxiety dimension as this dimension has distinct antecedents. Based on Table 1, it has been found that general AI anxiety is investigated with the highest number of antecedents, followed by job replacement anxiety and, subsequently, AI ethical anxiety, which has the fewest antecedents, suggesting that this dimension remains underexplored. These antecedents operate across different levels, including organizational, team, and individual levels although their relevance differs based on the focal dimension of AI anxiety. This indicates that the selection of antecedents is not uniform but aligned with the conceptual nature of each dimension of AI anxiety.

##### 2.2.3.2 Mediating variables

The mediating variables are classified by the AI anxiety dimension as this dimension has distinct mediators. Based on Table 1, it has been found that general AI anxiety is investigated with the highest number of mediator variables, followed by AI job replacement anxiety and AI learning anxiety, which are investigated with an equal number of mediator variables, while collective AI anxiety was investigated with only three mediator variables. These antecedents also operate across different levels, including organizational, team, and individual levels, although their relevance differs based on the focal dimension of AI anxiety. This indicates that the selection of mediating variables is not uniform but aligned with the conceptual nature of each dimension of AI anxiety.

##### 2.2.3.3 Moderating variables

The moderating variables are classified by AI anxiety dimension as each dimension has distinct moderators. Based on Table 1, it has been found that general AI anxiety is investigated with the highest number of moderator variables, followed by AI job replacement anxiety. Then, AI learning anxiety was investigated with the lowest number of moderator variables. These moderators also operate across different levels, including organizational and individual levels, although their relevance differs based on the focal dimension of AI anxiety. This indicates that the selection of moderating variables is not uniform but aligned with the conceptual nature of each dimension of AI anxiety.

##### 2.2.3.4 Outcome variables

The outcome variables are classified by AI anxiety dimension as each dimension has distinct outcome variables. Based on Table 1, it has been found that general AI anxiety is investigated with the highest number of outcome variables. This is followed by AI job replacement anxiety and AI learning anxiety. Three dimensions of AI anxiety, namely, AI opacity anxiety, AI privacy anxiety, and AI bias anxiety, were investigated with only two outcome variables. Finally, AI configuration anxiety and collective AI anxiety have the lowest number of outcome variables. These outcomes also operate across different levels, including organizational, team, and individual levels, although their relevance differs based on the focal dimension of AI anxiety. This indicates that the selection of outcome variables is not uniform but aligned with the conceptual nature of each dimension of AI anxiety.

#### 2.2.4 Methodology (M)

This section complements the TCCM synthesis by outlining the methodological approaches used in prior research on AI anxiety, with a particular focus on quantitative studies and their design, measurement, and data analysis strategies.

The methodological approach and the techniques for examining the relationships in AI anxiety research based on the reviewed articles are highlighted in this section. While the TCCM analytical framework allows including qualitative and quantitative studies, this study concentrates on quantitative studies for many reasons. First, the latter are used to achieve the current study’s objective and to address its research questions. As this study concentrates on synthesizing empirically examined relationships, including mediating and moderating mechanisms, quantitative studies are appropriate in this situation. Second, to identify the statistical techniques that measure these relationships, a quantitative approach should be employed. Finally, although the qualitative studies have valuable insights, their synthesis falls outside the scope of the present review. Therefore, to maintain coherent methodological consistency, this SLR focuses on reviewing previous studies that adopt a quantitative approach.

**Figure 5:**
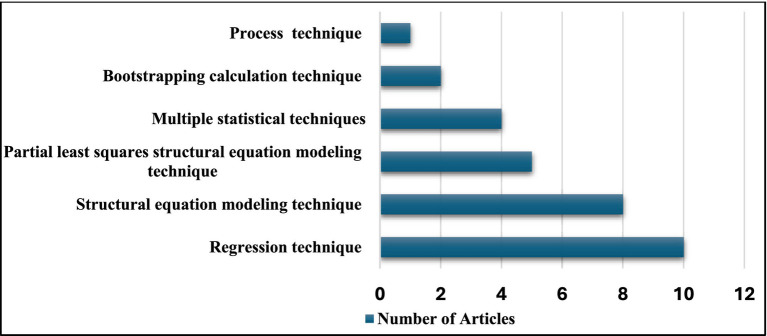
Frequency of statistical analysis techniques in AI anxiety studies.

This review found that the highest statistical technique used in the previous studies was the regression technique, which was applied in 10 studies, followed by the structural equation modeling technique (SEM), which was applied in eight studies. Then, partial least squares structural equation modeling (PLS-SEM) ranked third and was applied in five studies. Applying multiple statistical techniques ranked fourth and was applied in four studies, by integrating two of these techniques in each study: the path technique and the bootstrap resampling technique, the regression technique and PLS-SEM, and an artificial neural network technique (ANN), the regression technique, and Pearson’s correlation technique. The bootstrapping calculation technique ranked fifth and was applied in two studies. Finally, the process technique was the least frequently used statistical technique. Figure 5 shows the ranks of the statistical techniques and the number of reviewed studies that adopt each technique.

These methodological insights feed into the TCCM analytical framework by connecting quantitative approaches to the examined constructs, mediating and moderating mechanisms, outcomes, and the theoretical perspectives applied across studies.

## 3 Discussion

This research analyzed 30 empirical studies regarding AI anxiety by applying the SPAR-4-SLR procedure and the TCCM analytical framework. It provides structured synthesis by highlighting its theoretical perspectives, contextual differences, construct characteristics, and the types of quantitative statistical techniques applied.

From a theoretical perspective, this review found 15 theories used to investigate AI anxiety dimensions. SCR and COR are the dominant theories. This reflects that AI anxiety is primarily conceptualized through two complementary lenses: a cognitive–learning perspective and a resource-based stress perspective. This dominance can be attributed to the strong alignment between these two theories and the nature of AI anxiety. These theories can clarify the cognitive perceptions of AI and perceived threats to valuable resources such as job security and skills. These theories are also known for their suitability to be applied across individual and organizational levels, which might be another explanation for their widespread use. Another key point, SCR and COR theories were employed in the reviewed articles independently across different studies based on the objective of each study and the nature of the variables under each study. Another critical point is that SCT and COR are applied in several contexts (countries and industries). However, it has been found that they are applied more frequently in healthcare settings and in emerging economies such as China and Türkiye. These contexts are not theoretically homogeneous as other frameworks are also applied. Thus, this reflects that the nature of the relationship between theory and context in this systematic review can be described by tendencies instead of exclusivity.

Regarding the context, the highest number of reviewed articles was applied in China and Türkiye within the healthcare sector. This concentration may be explained by the rapid adoption of AI technologies and the high investment in AI research and development in these contexts. In addition, the focus in the healthcare sector can be explained by the nature of medical practices that require AI integration. However, the high frequency of the studies in these settings might bias the literature. In other words, these studies treated AI anxiety as a threat, especially within the healthcare sector, which has a high-pressure work environment. As a result, the positive side of AI anxiety might be underexplored, which limits the generalizability of the findings.

These theoretical and contextual patterns, with the construct characteristics identified in the previous section, clarify that the relationships in the AI anxiety dimensions literature are not isolated. Instead, it follows recurring explanatory logics. Based on these findings, the following section develops a mechanism-based framework that explains how antecedents, mediators, and outcomes interact across several AI anxiety dimensions under varying contextual conditions.

Building on the patterns identified in the characteristics section, the findings of these reviewed articles can be classified into three mechanism-based pathways: cognitive appraisal mechanism, resource-threat mechanism, and capability-based mechanism that explain how AI anxiety dimensions are investigated through several constructs and contexts.

Regarding the cognitive appraisal mechanism, it is adopted in studies that concentrate mostly on general and learning AI anxiety. In this pathway, organizational, technological, and individual antecedents shape employees’ views in terms of AI, which are processed via cognitive and affective mediators that impact behavioral outcomes such as adoption and innovation. SCT and SOR, which concentrate on evaluation processes by individuals toward AI, are aligned with this kind of mechanism.

The resource-threat mechanism is adopted in studies that concentrate mostly on job replacement anxiety. In this pathway, job insecurity and perceived technological substitution antecedents induce individuals’ anxiety feelings via emotional and strain-related mediators, such as emotional exhaustion, that lead to negative behavioral outcomes such as withdrawal behaviors. COR, which explains how perceived resource loss leads to stress and negative outcomes, is aligned with this kind of mechanism.

In relation to the capability-based mechanism, it is adopted in studies that concentrate mostly on learning and ethical dimensions of AI anxiety. In this pathway, perceived skill gaps and technological complexity impact anxiety via capability-related mediators, such as self-efficacy. This leads to adaptive or resistant responses. SCT, which explains the learning-oriented process and how people see themselves, impacts their ability to deal with technological change.

Finally, most of the reviewed studies used the regression technique as a quantitative statistical tool, while the least utilized technique was process. These findings reveal a clear imbalance within the analytical framework. The regression technique was applied more, which may be explained by its ease of application and its suitability for analyzing simple research models. Thus, the nature of the study affects the selected statistical technique.

Overall, the above discussion reflects how theories, constructs, contexts, and methods do not operate separately but rather interdependently to enhance the development and understanding of the several dimensions of AI anxiety, which has led to organized but diverse research.

This SLR provides two essential implications. First, it provides a comprehensive framework for AI anxiety dimensions for the research society by discussing AI anxiety from several angles. Second, it assists practitioners by realizing the AI anxiety phenomenon and its antecedents and consequences to create comprehensive strategies to deal with this issue.

## 4 Research limitations and future research directions

This review has two kinds of limitations. First, limitations related to the reviewed studies.

Despite the fact of the importance of the role of the environmental, institutional, and diffusion theoretical perspective, the number of applied theories that belong to this perspective is limited. Therefore, it is recommended to apply several institutional theories to highlight AI anxiety, such as sociotechnical systems theory (Emery and Trist, 1960; Trist and Bamforth, 1951) and Legitimacy Theory (Dowling and Pfeffer, 1975; Suchman, 1995). By using institutional theories as a theoretical foundation for investigating AI anxiety, it is essential to examine the antecedents of AI anxiety from an organizational AI governance perspective. Due to the transformation toward adopting AI, there is a need to examine the variables that are related to organizational AI governance structures. Furthermore, based on the review findings, most of the reviewed studies have been applied in China and Türkiye in the healthcare sector. Thus, future research is recommended to examine several AI anxiety types across diverse cultural and organizational contexts as there is a continuous trend to apply AI technologies worldwide and to have the ability to generalize the results. In addition, most of the reviewed studies examine individual-level constructs and their impact on several AI anxiety types. However, the entire picture should be investigated as employees’ responses to AI are also shaped by broader structural and institutional factors. Therefore, future research needs to adopt multi-level approaches to address this gap by investigating the effect of some constructs, such as organizational culture, organizational AI readiness, AI Governance, and ethical climate, on several dimensions of AI anxiety. Moreover, most of the reviewed studies primarily focus on general AI anxiety, resulting in a limited examination of its specific dimensions. This imbalance is notable given the important role that these distinct dimensions of AI anxiety play in shaping employee experiences within workplaces that adopt AI technology. Therefore, future research is highly recommended to highlight several AI anxiety dimensions, such as AI job replacement, AI learning anxiety, AI configuration anxiety, and collective AI anxiety. Finally, applying longitudinal studies and comparison studies will lead to valuable findings, particularly by using suitable quantitative statistical techniques such as SEM and multi-group analysis that allow for testing the hypotheses together in a unified model instead of testing them separately.

Second, there are some limitations regarding the review process of the current systematic review. Although the current review systematically mapped the literature using the TCCM analytical framework, several issues should be investigated by applying problem-driven design in the future. In addition, this review is limited to studies in WOS and Scopus indexed in SSCI and SCIE. These databases are widely recognized for their high-quality and peer-reviewed publications. While this approach ensures the reliability and rigor of the included studies, future research could extend the scope by including additional databases to provide a more comprehensive perspective and enable comparison with the findings of this review. These findings show that there is a limited understanding regarding the consequences of several AI anxiety dimensions, which are important factors during the artificial intelligence era across different work contexts. Finally, future research should move beyond descriptive synthesis toward a deep analysis to test mechanism-based pathways, aligning theories with constructs and contexts, and examining underexplored dimensions of AI anxiety.

## Data Availability

The original contributions presented in the study are included in the article/supplementary material, further inquiries can be directed to the corresponding author.
